# American Institute of Homœopathy

**Published:** 1852-10

**Authors:** 


*American Institute of Homœopathy.*—The proceedings of this body at their session in Baltimore, have been published. The whole number of members, as appears by their catalogue, which includes both the living and the dead, is 271. This aggregate is made up from the whole country, from Canada to California, and including both with the several States of the Union. As all the tribe in New York, of whom we have any knowledge, even the practitioners of Dentistry, fraternizing with them, are found in the list, we suppose that it is safe to infer that this sum total of 271 is the extent of "Homœopathic practitioners," who can be counted up on this continent.

As confirmatory of the estimate of the statistics of the sect, a few other data are found in this document which we subjoin.

In the city and county of Philadelphia, there are said to be *sixty* Homœopathic practitioners, and it is claimed that these are patronized by 90,000! one fifth of the population, which is rated at 450,000. A very modest pretension, certainly.

From Massachusetts, it appears that there are *fifty-nine* in the commonwealth, who, we are told, are all doing "a *paying* business" in Homœopathy; which is certainly high praise, for the Yankees are proverbial for "minding the main chance," and it is not unprofessional in the craft everywhere to imitate them.

The only other statistical items which can be gleaned from this document, are that, incidentally, mention is made of "4 Homœopaths in New Hampshire, 3 in Maine, 4 in Rhode Island, and 3 in other States," meaning New England, we suppose. But for greater accuracy, and for the reason that we would rather overrate than underrate the forces of the tribe, we refer to the general catalogue, by which it appears that the ratio for the different States stands thus, viz:—

New York, 83; Pennsylvania, 47; Massachusetts, 32; Ohio, 19; New Jersey, 14; Maine, 12; Connecticut, 10; Rhode Island, 10; New Hampshire, 7; Maryland, 7; California, 4; Louisiana, 3; Texas, 2; District of Columbia, 2; Alabama, 2; Virginia, 2; Illinois, 2; Wisconsin, Georgia, Delaware, and Canada, each 1; and a few others not located. Such is the classification of all the members of this National Society, which no doubt records the names of all in the country whom they recognize, good, bad, and indifferent, for many of these are certainly indifferent enough. If a similar enumeration of Physicians were made, there would be found five times as many in New York city alone, as there are Homœopaths in the whole country.

Nothing demonstrates the insignificance of the sect more clearly than these statistics furnished by themselves. And if they could only be required to show their colors in every place, by announcing themselves as Homœopaths on their signs and cards, as honesty demands, the public could then discriminate between them and physicians, and the line of distinction being thus drawn, they would be impotent, even though as numerous as the locusts of Egypt.—*N. Y. Med. Gaz.*

